# The Portal Vertex of KSHV Promotes Docking of Capsids at the Nuclear Pores

**DOI:** 10.3390/v13040597

**Published:** 2021-03-31

**Authors:** Daniela Dünn-Kittenplon, Asaf Ashkenazy-Titelman, Inna Kalt, Jean-Paul Lellouche, Yaron Shav-Tal, Ronit Sarid

**Affiliations:** 1 The Mina and Everard Goodman Faculty of Life Sciences, Bar-Ilan University, Ramat Gan 5290002, Israel; danadk8@gmail.com (D.D.-K.); asaf.ash1@gmail.com (A.A.-T.); Inna.Kalt@biu.ac.il (I.K.); Yaron.Shav-Tal@biu.ac.il (Y.S.-T.); 2 Department of Chemistry, Bar-Ilan University, Ramat Gan 5290002, Israel; Jean-Paul.M.Lellouche@biu.ac.il; 3 Advanced Materials and Nanotechnology Institute, Bar-Ilan University, Ramat Gan 5290002, Israel

**Keywords:** Kaposi’s sarcoma-associated herpesvirus (KSHV), open reading frame 43 (ORF43), portal, nuclear pore complex (NPC), capsid vertex-specific component (CVSC)

## Abstract

Kaposi’s sarcoma-associated herpesvirus (KSHV) is a cancer-related herpesvirus. Like other herpesviruses, the KSHV icosahedral capsid includes a portal vertex, composed of 12 protein subunits encoded by open reading frame (ORF) 43, which enables packaging and release of the viral genome into the nucleus through the nuclear pore complex (NPC). Capsid vertex-specific component (CVSC) tegument proteins, which directly mediate docking at the NPCs, are organized on the capsid vertices and are enriched on the portal vertex. Whether and how the portal vertex is selected for docking at the NPC is unknown. Here, we investigated the docking of incoming ORF43-null KSHV capsids at the NPCs, and describe a significantly lower fraction of capsids attached to the nuclear envelope compared to wild-type (WT) capsids. Like WT capsids, nuclear envelope-associated ORF43-null capsids co-localized with different nucleoporins (Nups) and did not detach upon salt treatment. Inhibition of nuclear export did not alter WT capsid docking. As ORF43-null capsids exhibit lower extent of association with the NPCs, we conclude that although not essential, the portal has a role in mediating the interaction of the CVSC proteins with Nups, and suggest a model whereby WT capsids can dock at the nuclear envelope through a non-portal penton vertex, resulting in an infection ‘dead end’.

## 1. Introduction

Kaposi’s sarcoma-associated herpesvirus (KSHV), also known as human herpesvirus-8 (HHV-8), is a cancer-related human virus, which is classified as a member of the *Gamma-2-herpesvirinae* subfamily. Infection with KSHV is etiologically linked to Kaposi’s sarcoma (KS), primary effusion lymphoma (PEL), and multicentric Castleman’s disease (MCD). It is also associated with KSHV-inflammatory cytokine syndrome (KICS), which clinically resembles MCD yet has unique pathologic features [1,2,3,4,5,6].

Like all herpesviruses, the KSHV proteinaceous icosahedral capsid is composed of 955 units of the major capsid protein (MCP) open reading frame (ORF) 25, arranged in 150 hexons and 11 penton vertices. The 12th vertex, is the portal vertex, a conserved herpesviral ring-like dodecameric structure comprised of the portal ORF43 protein subunit, which forms a channel through which the viral genome is inserted into newly assembled capsids, and released into the host cell nucleus [7,8,9,10,11,12,13,14,15]. The capsid is coated with a thick protein network, termed the tegument, which is wrapped within a phospholipid envelope that is decorated with viral glycoproteins. The tegument is composed of an outer layer, which is relatively amorphous, and an inner, finely-organized layer, which extends from the capsid vertices, and is, thus, termed capsid vertex-specific component (CVSC) or capsid-associated tegument complex (CATC). The KSHV CVSC, composed of ORF19, ORF32, and ORF64 (pUL25, pUL17, and pUL36 homologs of HSV-1, respectively), interacts with the capsid triplex ORF62 and ORF26 heterotrimer, with the MCP ORF25, and possibly with the small capsid protein (SCP) ORF65 near the penton vertices, as well [8,16,17]. Capsid assembly, which involves the scaffold protein ORF17.5, produces three types of mature capsids: A-capsids, which lack scaffold protein and do not contain DNA, B-capsids containing various quantities of the scaffold protein and no DNA, and C-capsids containing viral DNA genome and lacking scaffold protein. Only C-type capsids have the potential to generate productive infection [7,18,19,20].

Cryo-electron microscopy has recently provided high-resolution atomic structures of the portal vertex and the CVSC of KSHV [8,21], as well as of HSV-1 [9,10,17,22], HSV-2 [23,24,25], EBV [26,27], and VZV [28]. These models have shown that the KSHV portal vertex is enriched with the CVSC tegument complexes, and unlike the high occupancy of CVSC in all vertices of HSV-1 and HSV-2 capsids, CVSC complexes on the other 11 vertices of the KSHV capsids were only partially and flexibly occupied, reaching an average of approximately 30% occupancy. It was also shown in HSV-1 and HSV-2, that C-capsids have higher CVSC occupancy, probably due to higher affinity of the CVSC complex to the C-capsid vertices than to A- and B-capsids [17,23,25,28]. In addition to the CVSC proteins, the KSHV portal vertex has an outward protruding cap on the end of the genomic DNA. This cap, which appears to maintain the packaged viral DNA genome within mature capsids, is thought to be composed of ORF19 and possibly other tegument protein/s [8], and is similar in HSV-1 [9,10,17] and HSV-2 [23]. In this regard, the terminal DNA, which is located adjacent to the portal cap, may also function as a structural motif. Despite the high occupancy of the CVSC on the penton vertices in HSV-1, and in contrast to their low occupancy in KSHV, the HSV-1 CVSC protein pUL17 (KSHV ORF32 homolog) has a greater affinity for the portal vertex as compared to other penton vertices. Furthermore, a 30Å outward displacement of the portal complex upon DNA packaging in C-capsids has been described in HSV-1 and HSV-2, and this may result in conformational changes of the CVSC complex at this vertex. These occupational and conformational characteristics may provide the CVSC at the portal vertex with unique functions such as recruitment of the terminase complex machinery toward packaging of the viral DNA within newly assembled capsids, binding the cap proteins to retain the highly compressed DNA in the capsid, and docking at the nuclear pores [17,23].

**Figure 1:**
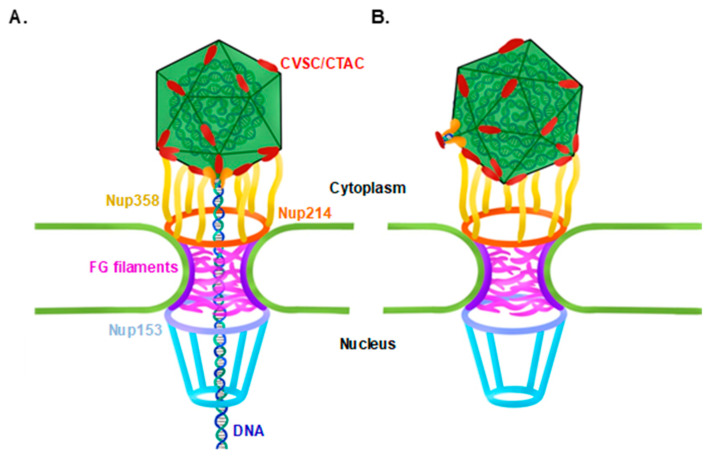
Models for capsid docking at the nuclear pore. Proper docking orientation of the capsid with the portal vertex facing the nuclear pore enables ejection of the viral DNA to the nucleus through the portal, across the nuclear pore complex (NPC). Nuclear docking is mediated by the capsid vertex-specific component (CVSC; also termed capsid-associated tegument complex (CATC)) (**A**). Theoretical model for improper docking orientation, with one of the 11 penton vertices facing the nuclear pore, prevents DNA release and, thus, leads to a ‘dead-end’ infection (**B**). Nucleoporins (Nups) 358, 214, and 153, Phenylalanine-Glycine (FG) filaments.

All herpesviruses inject their genomic DNA through the nuclear pore complex (NPC) following capsid trafficking towards the nucleus and upon docking at the nuclear envelope. Docking of HSV-1 at the NPC is mediated by the tegument proteins pUL25 (KSHV ORF19 homolog), which binds Nup214 situated on the cytoplasmic NPC surface [29,30], and pUL36 (KSHV ORF64 homolog), which binds Nup358 localized on the cytoplasmic NPC filaments [31,32]. These interactions likely facilitate proper alignment of the capsid at the NPC for viral genome ejection (Figure 1A). However, improper docking, through one of the vertices that is not the portal vertex, as shown in Figure 1B, may prevent delivery of the viral DNA genome through the NPC [33]. Upon proper docking at the nuclear pores, the viral genome is rapidly released through the portal into the nucleoplasm. Ejection of the genome from the capsid is promoted by the high internal pressure of tens of atmospheres, which forcefully overcomes the NPC permeability barrier to enter into the nucleus [34,35,36,37]. Whether the docking of the capsid at the NPC is a stochastic event or driven to the correct orientation by the structure of the unique portal vertex is currently unknown. Furthermore, it is not clear whether the DNA genome is required, as a structural element, for docking [8,9,10,38].

In the present study, we employed KSHV virions that lack the portal structure, and examined their docking at the NPC upon infection as compared to wild-type (WT) capsids. We report that mutated portal-null capsids dock at NPCs, yet at a lower extent than WT capsids. This suggests that docking of KSHV capsids at the NPCs could occur at an inappropriate orientation, with the portal not facing the NPCs, resulting in their inability to deliver the viral DNA.

## 2. Materials and Methods

### 2.1. Cell Cultures and Viruses

Renal cell carcinoma lines, SLK and iSLK (SLK cells containing Tet-on transactivator and RTA expression cassette) (kindly provided by Don Ganem, Howard Hughes Medical Institute, UCSF, San Francisco, CA, USA and Rolf Renne, University of Florida, Gainesville, FL, USA) [39], were grown in Dulbecco’s modified Eagle’s medium (DMEM) supplemented with 10% fetal calf serum (FCS), 50 IU/mL penicillin, and 50 µg/mL streptomycin (Biological Industries, Kibbutz Beit Haemek, Israel). iSLK cells were also supplemented with 250 µg/mL G418 (A.G. Scientific Inc., San Diego CA) to maintain the Tet-on transactivator, and with 1 µg/mL puromycin (A.G. Scientific Inc.) to maintain the K-RTA expression cassette. BAC16-infected iSLK cells were supplemented with 600 µg/mL hygromycin B (A.G. Scientific Inc.) to maintain KSHV episomes. Human U2OS osteosarcoma cells were maintained in low-glucose DMEM supplemented with 10% FCS, 50 IU/mL penicillin, 50 µg/mL streptomycin, and 2 mM L-glutamine (Biological Industries, Kibbutz Beit Haemek, Israel). All cells were grown at 37 °C in a humidified atmosphere with 5% CO_2_.

The BAC16 (a kind gift of Prof. Jae Jung) [40] clone containing the full-length WT KSHV genome encoding GFP and hygromycin resistance under the control of EF-1α promoter, and BAC16 ORF43-null mutant, which contains two stop codons in the *orf43* gene were previously described [13].

### 2.2. Lytic Reactivation of KSHV in iSLK Infected Cells and Virion Purification

KSHV-infected iSLK cells were treated with 1 μg/mL doxycycline and 1 mM n-Sodium butyrate (Sigma), in the absence of hygromycin, puromycin, and G418, to induce RTA transgene expression and lytic cycle reactivation. Supernatants containing virions were collected 96 h later, and cleared from cells and debris by two cycles of centrifugation (1000× *g* for 10 min at 4 °C) and filtration (0.45-μm cellulose acetate filters (Corning)). Supernatants were then centrifuged for 2 h at 40,000× *g* at 4 °C, and the pellet was resuspended in a small volume (approximately 40-fold reduced volume) of DMEM, overnight at 4 °C. Concentrated virions were collected and were either used to infect cells immediately or were stored at −80 °C.

### 2.3. Virus Quantification

To quantify the infectious KSHV virions, SLK cells were infected with different volumes of concentrated virions 24 h after seeding, by 1-h spinoculation at 1500× *g* at 25 °C, followed by 1-h incubation at 37 °C. The infected cells were incubated with medium supplemented with 5% FCS. Cells were trypsinized (Biological industries) 48 h post-infection (PI), washed with PBS, and fixed in 2% formaldehyde in PBS for 20 min at 4 °C. Cells were washed with PBS, and percent GFP-positive cells was quantified by fluorescence-activated cell sorting (FACS (Gallios; Beckman Coutler)) analysis, followed by FlowJo software analysis. The multiplicity of infection (MOI) was calculated according to the % GFP-positive cells, and the number of cells, by using the equation: MOI = −ln (fraction of GFP negative cells), and was subsequently used to determine the number of infectious units.

Titer of ORF43-null virions, which do not contain viral DNA, was determined by using Western Blot analysis with purified virions. ORF43-null virions and WT virions at different volumes, were boiled with sample buffer (SB) for 10 min, and analyzed by SDS-PAGE followed by antibody detection of the small capsid protein ORF65 (a kind gift from Shou Jiang Gao) [41]. The intensities of the resulting bands were analyzed by ImageJ software, and the relative titer of the mutated virions was determined in comparison to WT virions of known titer. Titers of ORF43-null virions, were also determined by using immunofluorescence assay (IFA) with ORF65 antibody, and de-novo infection. Z-stacks option (Zs) were collected and the function of ‘maximun projection’ in LASX software (Leica) was used to project all the Zs followed by manual counting of the virions.

### 2.4. De Novo Infection

SLK or U2OS cells were infected with KSHV in the presence of 8 µg/mL polybrene by spinoculation (centrifugation at 1500× *g* at 4 °C for 60 min), which was followed by replacement of the medium with fresh medium containing 5% FCS and incubation at 37 °C for the indicated time.

### 2.5. Immunofluorescence

SLK or U2OS naïve cells were seeded on cover slips in a 24-well plate. After 24 h, cells were infected with purified virions by spinoculation at 1500× *g* for 1 h at 4 °C. Cells were washed with medium and maintained with the appropriate medium supplemented by 5% FCS. At the indicated time points post-infection, cells were washed twice with PBS, fixed with 4% para-formaldehyde in PBS for 20 min at room temperature, washed with PBS, and permeabilized and blocked with 0.2% Triton X-100 and 1% BSA (Sigma) in PBS for 30 min at room temperature. In some experiments, exposed nuclei were generated by treatment with 0.1% Triton X-100 for 5 min on ice before fixation. The exposed nuclei were subjected to salt treatment immediately after Triton X-100, by three cycles of 5-min incubation with 0.5 or 1 M NaCl:KCl 53:1, followed by three washes with PBS, before fixation. Slides were then incubated for 1 h at room temperature with a primary antibody (rabbit anti-Nup214, Nup153, Nup358 or Lamin A (Abcam)) followed by incubation for 1 h at room temperature with a conjugated anti-rabbit secondary antibody (Cy3 or Alexa Fluor 647 (Jackson ImmunoResearch Laboratories, Inc., West Grove, PA, USA)). Cells were then incubated for 2 h at room temperature with mouse anti-ORF65 [41] for capsid staining, followed by 1 h incubation with a secondary conjugated anti-mouse antibody (Rhodamine, Alexa Fluor 488 or Alexa Fluor 647 (Jackson ImmunoResearch Laboratories, Inc., West Grove, PA, USA)). To stain the nuclei, cells were incubated for 30 min at room temperature with 0.05 µg/mL Hoechst dye (Sigma) in PBS.

Confocal imaging employed #1 coverslips (Bar-Naor) that were mounted with 90% glycerol, 9% PBS, 1% n-Propyl Gallate (Sigma) anti-fade solution, while stimulated emission depletion (STED) microscopy employed high-precision #1.5 coverslips (Thermo Scientific) that were mounted with 80% glycerol with p-phenylenediamine antifade (Sigma) solution. Cells were imaged by confocal or STED microscopy, using a Leica SP8 STED confocal microscope, with 100× 1.4 NA lens. Dual-color STED experiments were performed by between-line sequential imaging using the 660 nm depletion laser set at 90% (slider) of laser power for Alexa Fluor 488, and 50% slider for Rhodamine Red-x or Cy3 for accurate spatial imaging. Images were deconvolved with Huygens Professional (Scientific Volume Imaging) using the CMLE algorithm, with a signal-to-noise ratio of 12 and 40 iterations, using the Huygens STED module.

### 2.6. Inhibition of Nuclear Export by WGA

U2OS cells were incubated for 5 min, at 37 °C, with 5 µg/mL Cy5-wheat germ agglutinin (WGA) (Invitrogen), together with 30 µg/mL digitonin (Sigma), in medium supplemented with 5% FCS, 30 min after infection [42]. After WGA treatment, cells were washed three times with medium, and maintained with medium supplemented with 5% FCS. After 4.5 h, nuclei were exposed by Triton X-100 for 5 min on ice, fixed and stained, as described in Section 2.5.

## 3. Results

### 3.1. KSHV Capsids That Lack the Portal Structure Accumulate at the Nuclear Envelope at a Lower Extent than Wild-Type Capsids

**Figure 2:**
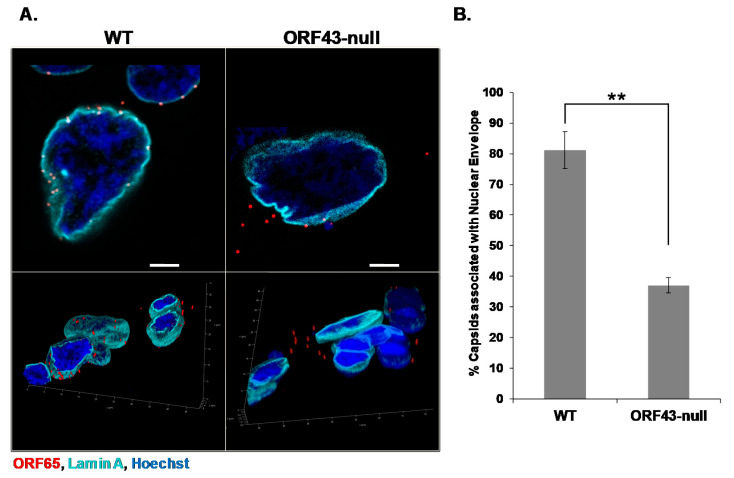
Open reading frame (ORF) 43-null Kaposi’s sarcoma-associated herpesvirus (KSHV) capsids display relatively lower extent of association with the nuclear envelope compared to wild-type (WT) capsids. SLK cells were infected with WT or ORF43-null KSHV BAC16 virions. Six hours post-infection, cells were fixed, and capsids were detected with an antibody to the small capsid protein ORF65 and a Rhodamine-conjugated anti-mouse secondary antibody (red), while the nuclear envelope was observed using an antibody to Lamin A and an Alexa Fluor 647-conjugated anti-rabbit secondary antibody (cyan). Three-dimensional images were acquired by Z-stack with the super Z galvanometer substage, and 3D reconstruction with LASX software (Leica) (**A**). 1330 cells, containing 7890 WT capsids, and 2231 cells, containing 4290 ORF43-null capsids were counted in three independent experiments. The percentage of WT and ORF43-null capsids associated with the nuclear envelope is presented. Data are expressed as mean ± SD. ** *p* = 0.0023 (*p* < 0.01) (**B**). Scale bars = 5 µm.

The portal ring serves as a gate for herpesviral genome release from the capsid through the NPC. However, it is not known whether capsid docking at the nuclear pore requires the portal, or whether any capsid vertex may be recognized regardless of the portal structure. To address this issue, we took the advantage of a recombinant clone of the KSHV genome, BAC16 ORF43-null, which fails to express the KSHV portal protein ORF43 due to two stop codon mutations, hence, lacking the portal vertex. We have previously shown that this recombinant genome generates virions that are not loaded with genomic viral DNA [13]. Using the spinoculation method, we infected SLK cells with BAC16 wild-type (WT) or BAC16 ORF43-null virions and fixed the cells 6 h post-infection. Both WT and ORF43-null capsids were detected by immunofluorescence with an antibody to the small capsid protein ORF65, while the nuclear envelope was marked with an antibody to Lamin A. The capsids were counted and classified according to their localization as co-localized with Lamin A or as cytoplasmic. A small number of capsids, detected ‘on’ the nucleus, were neglected because their vicinity to the nuclear envelope was unclear. As shown in Figure 2, a fraction of the ORF43-null KSHV mutant capsids were associated with the nuclear envelope, yet this fraction was significantly lower than that observed with WT capsids. Similar results, revealing a clear difference in the fraction of envelope-associated capsids between WT and ORF43-null mutant, were obtained in U2OS cells. Furthermore, similar results were obtained when cells were infected with different multiplicities of infection (MOIs), ranging from 1 to 10 infectious units (IUs) per cell (data not shown). These findings suggest that the reduced association of ORF43-null capsids with the nuclear envelope, compared with WT capsids, is not cell type-specific and is not affected by the MOI. Furthermore, our results suggest that the portal has a direct or indirect role in mediating the interaction of the CVSC tegument proteins with the NPCs.

### 3.2. The Association Extent of ORF43-Null Capsids with the Nuclear Envelope Do Not Increase at Later Time Points Post-Infection

**Figure 3:**
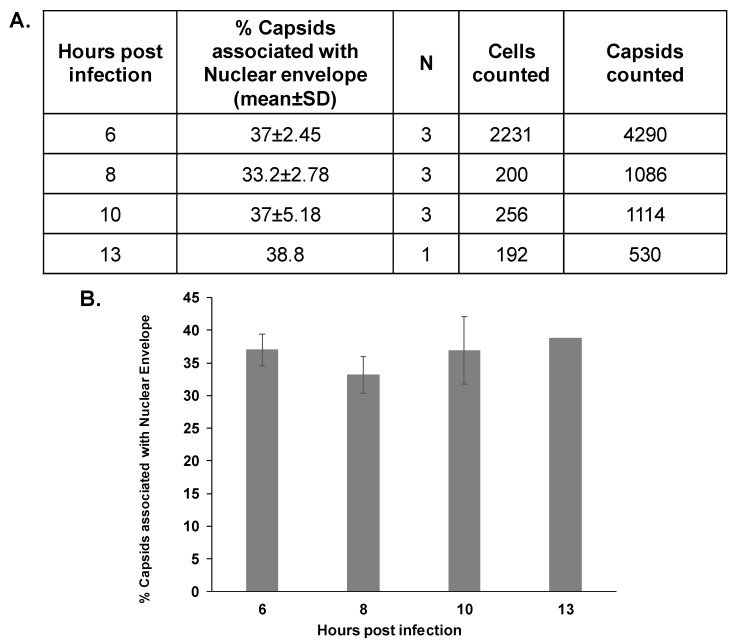
The proportion of ORF43-null capsids that are in proximity to the nuclear envelope does not increase over time. SLK cells were infected with BAC16 ORF43-null virions. Cells were fixed 8, 10, and 13 h post-infection, and virions were detected with an antibody directed against the small capsid protein ORF65, and the nuclear envelope was observed with an antibody to Nup214. ORF43-null capsids were counted. Then, 200, 256, and 192 cells containing 1086, 1114, and 530 ORF43-null capsids that were fixed 8, 10, 13 h post-infection, respectively, were evaluated. Results presented at 8 and 10 h post-infection are from three independent experiments, while one experiment was carried out for 13 h post-infection. Results presented for 6 h post-infection are the same experiments shown in Figure 2B (**A**). Plot showing the percentage of capsids bound to the nuclear envelope. Data are expressed as mean ± SD. No significant difference between the mean values by one-way ANOVA test (*p* = 0.397) (**B**).

The relatively lower fraction of ORF43-null capsids that were associated with the nuclear envelope, compared with WT capsids, could be due to slower trafficking kinetics of these virions within the cells toward the nucleus. Therefore, we infected SLK cells using spinoculation, and examined the association of ORF43-null capsids with the nuclear envelope at later time points following infection. Capsids were detected with ORF65 antibody, and the nuclear envelope was marked with an antibody to Nup214, which is found on the cytoplasmic side of the NPC (Figure 1). Capsid localization was classified as described above. As shown in Figure 3, no significant difference in the proportion of capsids that co-localized with the nuclear envelope was evident at the different time points. This suggests that the significant difference between WT and ORF43-null viruses, in the percentage of the capsids that were in proximity with the nuclear envelope, is not due to slower entry or trafficking kinetics of the ORF43-null virions.

### 3.3. Co-Localization of WT and ORF43-Null Capsids with Nucleoporins

**Figure 4:**
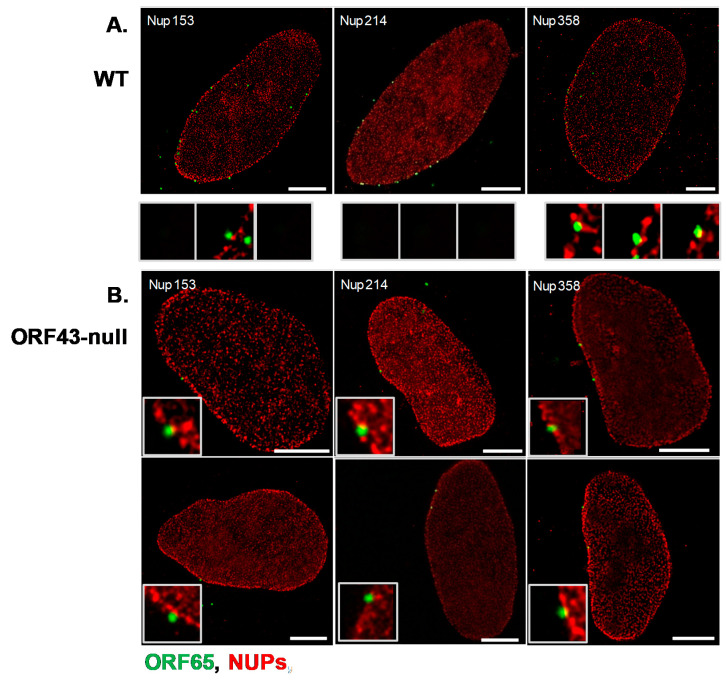
Co-localization of KSHV WT and ORF43-null capsids with Nup358, Nup214, and Nup153 detected by stimulated emission depletion (STED) microscopy. U2OS cells were infected with WT or ORF43-null KSHV. Six hours post-infection, cells were treated with 0.1% Triton X-100 for 5 min, to remove the cytoplasm, and fixed in 4% para-formaldehyde in PBS for 20 min. Capsids were detected with an antibody to ORF65 and a secondary anti-mouse conjugated to Alexa 488 (green), and co-localization with Nup214, Nup153, or Nup358 was examined with Nup-specific antibodies, and secondary anti-rabbit Cy3-conjugated antibodies (red); WT capsids (**A**), ORF43-null capsids (**B**). Scale bars = 5 µm.

Given the proximity of a fraction of ORF43-null capsids with the nuclear envelope, we next investigated whether the observed association of ORF43-null capsids with the nuclear envelope represents docking of capsids at the NPCs as previously reported for other herpesviruses. To track capsid docking at the nuclear pores, we used STED super-resolution microscopy. For this analysis, we infected U2OS cells with BAC16 WT or ORF43-null virions, and 6 h post-infection treated the cells with Triton X-100 to expose the nuclei. Cells were fixed, and capsids were detected with an ORF65 antibody along with antibodies recognizing Nup153, Nup214, or Nup358, representing different zones of the nuclear pore complex (NPC) [43] (Figure 1). As shown in Figure 4, ORF43-null capsids presented similar patterns of co-localization as WT capsids with all tested Nups. Both ORF43-null and WT capsids were interior relative to Nup358, which resides in the outer cytoplasmic side of the nuclear pore, indicating that the capsids were embedded on the cytoplasmic face of these pores. In addition, both capsids were partially co-localized with Nup214 residing in the cytoplasmic part of the NPC, and external relative to Nup153, which is located in the inner basket of the nuclear pore. These findings suggest that the observed association of ORF43-null capsids with the nuclear envelope reflects the docking of the capsids at the cytoplasmic side of the NPCs, in a manner similar to the docking of the WT capsids, and most likely involves one of the penton vertices.

### 3.4. High Salt Treatment Does Not Alter the Proportion of Bound Capsids to the Nuclear Envelope

**Figure 5:**
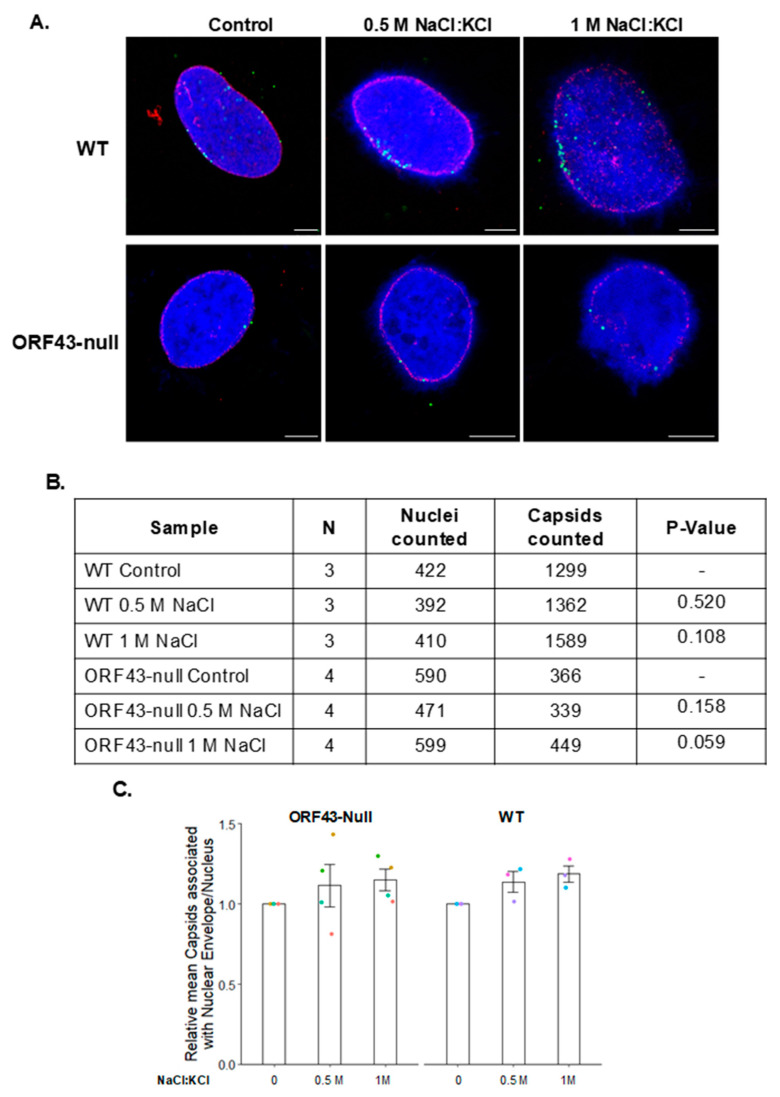
Docking of WT as well as ORF43-null KSHV capsids at the NPCs is not affected by high-salt treatment. U2OS cells were infected with WT or ORF43-null KSHV virions. Six hours post-infection, cells were treated with Triton X-100 to remove the cytoplasm, washed three times with 0.5 or 1 M salt (NaCl:KCl 53:1), and fixed. Capsids and Nup214 were detected by confocal microscopy, as described in Figure 3. Scale bars = 5 µm (**A**). The proportion of capsids that are in proximity with Nup214 was determined based on the indicated numbers of nuclei and capsids in three and four independent experiments for WT and ORF43-null virions, respectively. No significant difference between the mean values of both WT and ORF43-null virions was detected by one-sample *t*-test, indicating that the number of attached capsids did not decrease following high-salt treatments (**B**). Plot presenting the relative number of capsids associated with the nuclear envelope. Data are expressed as mean ± SD (**C**).

To determine whether there is a difference in the interaction strength between the WT and ORF43-null capsids with the NPCs, we examined the effect of high-salt treatment, which is known to disrupt weak interactions. We infected U2OS cells with WT or ORF43-null virions, and 6 h later subjected the nuclei to high salt treatment. Nuclei were fixed, and the capsids were stained with an antibody to ORF65, while the nuclear envelope was detected with antibody to Nup214. As shown in Figure 5, high salt treatment resulted in chromatin protrusions with spike-like structures, yet both WT and ORF43-null capsids remained associated with the nuclear envelope following 0.5 and 1 M salt treatments. This suggests that once either WT or ORF43-null capsids dock at the NPCs, they are strongly bound and do not detach even at high salt concentrations. Furthermore, it appears that like WT, the interaction of the ORF43-null capsids with NPCs is strong.

### 3.5. Export Inhibition Does Not Affect the Proportion of Nuclear Envelope-Associated Capsids

**Figure 6:**
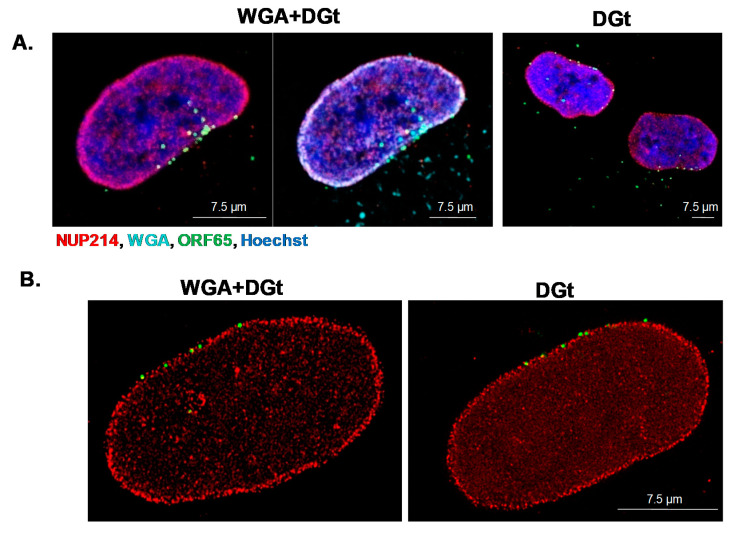

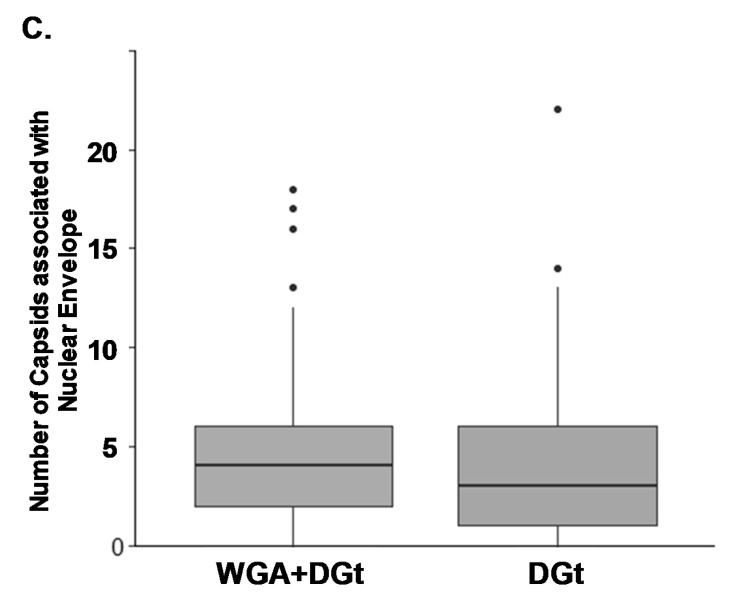
Export inhibition by wheat germ agglutinin (WGA) treatment does not interfere with capsid docking at the nuclear envelope. U2OS cells were infected with WT KSHV virions. After 30 min, cells were incubated for 5 min with 5 µg/mL Cy5-WGA (cyan) along with 30 µg/mL digitonin (DGt). Cells were then washed and further incubated for 4.5 h, and then treated with Triton X-100 to remove the cytoplasm. Capsids and Nup214 were detected as described in Figure 3 (**A**). The relative localization of the capsids and Nup214 was detected by STED microscopy, as described in Figure 4. Scale bars = 7.5 µm (**B**). 150 U2OS nuclei and 1630 capsids treated with Cy5-WGA and digitonin, and 108 U2OS nuclei and 1239 capsids treated with digitonin-only as a control, were inspected to determine the number of capsids associated with the nuclear envelope. The average number of capsids on the nuclear envelope was similar and no significant difference between WGA and control were found using Mann–Whitney *U*-test. *p* = 0.2115. Dots represent extreme values (**C**).

Wheat germ agglutinin (WGA) was previously shown to bind Phenylalanine-Glycine (FG) in NPCs and to inhibit export [44,45]. WGA and capsids interact with the nuclear pores from the cytoplasmic direction and high dose WGA (0.1–0.5 mg/mL) prevents the docking of capsids at the nuclear pores [36,46]. At a low dose (5 µg/mL), WGA was shown to block mRNA export [42,47,48]. We examined whether mRNA export inhibition with low dose WGA interferes with docking of the capsids at the nuclear pores. Cells were treated for 5 min with low dose Cy5-WGA (5 μg/mL) and digitonin 30 min post-infection with WT KSHV virions, and were then washed and further incubated for 4.5 h. Subsequently, the nuclei were exposed and capsids were detected by immunofluorescence microscopy. Cy5-WGA accumulated at the nuclear envelope (cyan), yet docking of the capsids at the nuclear envelope was not affected by WGA treatment (Figure 6). Further inspection using STED microscopy confirmed similar co-localization patterns of the capsids with Nup214 in cells that were treated with WGA, compared to control cells that were treated with digitonin (Figure 6B). Nuclei after WGA+digitonin treatment, were counted and compared to nuclei treated with digitonin only, to compare the membrane associated capsids, as shown in Figure 6C. We conclude that low dose treatment with WGA, which is known to interfere with mRNA export, does not affect the docking of KSHV capsids at the NPCs.

## 4. Discussion

Like all herpesviruses, the KSHV virions are composed of an icosahedral capsid, which contains a double-stranded DNA genome, coated with tegument proteins and wrapped within a phospholipid membrane decorated by viral glycoproteins. The icosahedral capsid has 11 penton vertices and one portal vertex, composed of 12 units of ORF43 protein, through which the viral DNA is inserted to newly assembled capsids and injected to the host cell nucleus during *de novo* infection. The capsid vertices are decorated with CVSC tegument proteins at different densities, including the tegument proteins ORF19, ORF32, and ORF64 (HSV-1 pUL25, pUL17, and pUL36 homologs, respectively). These tegument protein complexes are also located on the portal vertex, in which ORF19, and possibly other protein/s, serve as a cap for the packaged DNA [8,17].

Different virus families, which reproduce in the nucleus, employ various strategies for the delivery of the viral genome into cells (reviewed in [49,50,51,52,53]). For example, the adenovirus capsid docks at the NPC and disassembles through Nup214 interaction, causing displacement of the Nups, resulting in increased permeability of the NPC. Capsid binding to Kinesin-1 then destabilizes the capsid and enables the release of the dsDNA genome through the NPC [54,55]. The parvovirus capsid, which is 18–26 nm in diameter, is small enough to enter the NPC in its intact form, but does not use it. Instead, it binds the NPC and disrupts the nuclear envelope and nuclear lamina, followed by nuclear entry of the capsid through the resulting 100–200 nm gaps [56,57]. Simian virus (SV)-40 polyomavirus also disrupts the nuclear lamina and penetrates the nucleus through the endoplasmic reticulum (ER) or through the NPC, concurrently with capsid disassembly [58,59]. HIV reverse transcriptase transcribes its RNA genome to DNA in the cytoplasm, within the nucleocapsid core, while the DNA is inserted into the nucleus after the capsid dissociates, as part of a pre-integration complex containing the matrix and the integrase proteins that carry nuclear localization signal (NLS) motifs [60]. Influenza virus imports its viral ribonucleoproteins (vRNPs) to the nucleus as separate segments through the NPC using the NLS motif in the nucleoprotein, which is a part of the vRNP [61,62,63].

In herpesviruses, a crucial step in the infection cycle involves docking of the capsids at the nuclear pores and insertion of the viral DNA genome through the nuclear pores into the nucleus. The CVSC tegument protein complex mediates the interactions that promote docking of the capsid at the nuclear pore [34,49,50,51,52]. In analogy to HSV-1, and based on amino acid sequence and structural similarities, it is likely that the pUL25 homolog ORF19 interacts with Nup214, while the KSHV pUL36 homolog ORF64 interacts with Nup358. In addition, by analogy to HSV-1, DNA release into the nucleus is expected to require cleavage of ORF64 by an as yet unidentified protease [31]. Self-cleavage of ORF64 is possible, as its HSV-1 homolog pUL36 is a cysteine protease with deubiquitinating activity, and ORF64 is a known ubiquitin-specific protease [64,65]. As the VP24 protease encoded by HSV-1 cleaves pUL36, it is also possible that its KSHV homolog ORF17 cleaves ORF64 through its protease domain [31,66,67,68]. Enriched inner tegument CVSC complexes were detected in the portal vertex of KSHV, and unlike the high occupancy of these complexes in HSV-1 [10,16,21] and HSV-2 [23,25,69] C-capsids, the 11 other vertices in KSHV C-capsids are partially and flexibly occupied with the CVSC, reaching approximately one-third of the full occupancy [8]. It was also shown that C-capsids of HSV-2 and VZV have higher CVSC occupancy than A- and B-capsids [25,28].

In the present study, we aimed to elucidate the importance and significance of the portal structure for the docking of KSHV capsids at the nuclear pores. We employed KSHV portal ORF43-null virions, and demonstrate that the KSHV ORF43-null mutant exhibited a clear and consistent lower extent of association of the capsids with the nuclear envelope in comparison to WT capsids. As we did not detect increasing accumulation of the mutant ORF43-null capsids on the nuclear envelope at later times post-infection, the possibility of delayed trafficking of these capsids towards the nucleus was excluded. Yet, defective trafficking of ORF43-null virions toward the nucleus was not excluded.

The association of ORF43-null capsids with the nuclear membrane indicated that the CVSC tegument proteins of these portal-null capsids can associate with Nups of the NPC. Indeed, we found that the ORF43-null capsids co-localized with the NPCs in a similar pattern as WT capsids. Both capsids were somewhat embedded in Nup358, partially co-localized in the same plane with Nup214, which resides in the cytoplasmic domain of the nuclear pore, and partially protruding from the Nup153, which is located in the inner basket of the nuclear pore. We could not detect any difference in the localization pattern of the mutant ORF43-null capsids relative to the localization of the Nups, compared with the WT capsids. This suggests that KSHV capsids can dock at the NPCs via any penton vertex.

To determine whether there is a difference in the interaction strength between the WT and the ORF43-null capsids with the NPCs, we treated the nuclei with high salt concentrations to disrupt weak interactions. Surprisingly, the number of WT and ORF43-null capsids detected bound to the nuclear envelope was not affected following 0.5 and 1 M salt treatment, compared to untreated nuclei. This led us to conclude that once a capsid has abundant CVSC protein complexes on any penton vertex, which enable the interaction with the NPC, it is strongly bound and does not detach even in the presence of high salt concentrations. Because of the low occupancy of the penton vertices with CVSC, the binding probability of such a vertex facing the NPC is lower than that of the portal vertex, which is decorated with the CVSC complex and with an additional cap on the packaged DNA, and well exposed to dock at the nuclear pore [8,17].

As different CVSC occupancy, and different portal and CVSC structures, were found in C-capsids, in comparison to A- and B-capsids, it is possible that the packaged genomic viral DNA functions as a structural component, which enhances the docking probability of C-capsids through the portal vertex, whereas A- and B- capsids, as well as ORF43-null capsids that do not include ORF19 cap structure are expected to have lower docking extents [17]. Our results suggest that the preferred occupancy of the CVSC proteins at the portal vertex [8] leads to a preferred orientation of nuclear pore docking at the correct orientation, with the portal facing the nuclear pore. Incorrect orientation is also possible, though it occurs at a lower extent. These findings suggest that WT A- and B-capsids can also dock at the nuclear pores, although their CVSC complex may differ in composition and conformation from the portal vertex. Since it was found that there is a 30 Å outward shift of the portal complex in C-capsids, compared to the A- and B-capsids in HSV-1, and between C- and B-capsids in HSV-2, these differences in topology can also explain the different extents of docking between the capsid types [17,23].

We conclude that since the ORF43-null capsid also docks at the nuclear envelope, this interaction must occur via a regular penton vertex, and, thus, some of the WT C-capsids, probably bind incorrectly to the nuclear membrane through one of these 11 pentons, and not with the portal vertex facing the nuclear pores, as shown in Figure 1B. Such docking is expected to terminate the infection cycle. In addition, our findings suggest that the portal indirectly or directly mediates the interaction of the CVSC proteins with the Nups. The probability of docking with a penton vertex is unknown, yet the percentage of mutant ORF43-null capsids that were associated with the nuclear envelope can provide us a hint. As both WT and ORF43-null capsids remained associated with the NPCs following high salt treatment, it appears that like WT capsids ORF43-null is strongly bound to the NPCs. Whether full occupancy of the nuclear-docked penton vertex by CVSC complexes is required is not yet known. We have also excluded the possibility that inhibition of mRNA export with WGA (5 µg/mL), inhibits the docking of the capsids at the nuclear envelope [42,47]. Higher concentrations of WGA (0.1–0.5 mg/mL) were shown to inhibit infection, but probably through other pathways or mechanisms [44,45,46]. Our findings, which have not been previously reported for any other herpesvirus, suggest that orientation of capsids docking at the nuclear envelope represents a barrier during herpesvirus infection. Still, different herpesviruses may have different occupancy levels of the CVSC, which may alter the frequency of ‘dead-end’ docking of the capsids.
